# Supplementary material for: MetaRibo-Seq measures translation in microbiomes
Source: Nat Commun. 2020 Jun 29;11:3268. doi: 10.1038/s41467-020-17081-z (PMC7324362; doi:10.1038/s41467-020-17081-z)
Supplement: Supplementary file 10 — Supplementary Data 7 [file 41467_2020_17081_MOESM10_ESM.zip › File2/Confidence_High_Taxonomy/207398_out.krona.html]

Javascript must be enabled to view this page.

members
magnitude
magnitudeUnassigned
count
unassigned
taxon
rank

207398\_out

3

3
2
superkingdom

phylum
1239
3

class
186801
3

186802
order
3

1
31979
family

genus
1485
1


SRS024132\_contig\_number\_contig-100\_12.260655
1262781
species
1

541000
family
1

292632
genus
1

1
2302962
species

SRS1041140\_contig\_number\_21032

990719
family
1


SRS020869\_contig\_number\_24534
1
species
1930012
